# Heterogeneity of Dengue Illness in Community-Based Prospective Study, Iquitos, Peru

**DOI:** 10.3201/eid2609.191472

**Published:** 2020-09

**Authors:** William H. Elson, Robert C. Reiner, Crystyan Siles, Isabel Bazan, Stalin Vilcarromero, Amy R. Riley-Powell, Ania B. Kawiecki, Helvio Astete, Robert D. Hontz, Chris M. Barker, Gonzalo M. Vazquez-Prokopec, Amy C. Morrison, Thomas W. Scott, John P. Elder, Alan L. Rothman, Valerie A. Paz-Soldan

**Affiliations:** University of California Davis, Davis, California, USA (W.H. Elson, A.B. Kawiecki, C.M. Barker, A.C. Morrison, T.W. Scott);; University of Washington School of Medicine, Seattle, Washington, USA (R.C. Reiner);; US Naval Medical Research Unit No. 6, Lima and Iquitos, Peru (C. Siles, I. Bazan, S. Vilcarromero, H. Astete, R.D. Hontz, A.C. Morrison);; University of Sussex, Brighton, UK (A.R. Riley-Powell);; Tulane School of Public Health and Tropical Medicine, New Orleans, Louisiana, USA (A.R. Riley-Powell, V.A. Paz-Soldan);; Emory University, Atlanta, Georgia, USA (G.M. Vazquez-Prokopec);; San Diego State University, San Diego, California, USA (J.P. Elder);; University of Rhode Island, Providence, Rhode Island, USA (A.L. Rothman)

**Keywords:** dengue, dengue virus, viruses, epidemiology, cohort studies, community-based prospective study, heterogeneity, humans, Iquitos, Peru

## Abstract

Measuring heterogeneity of dengue illness is necessary to define suitable endpoints in dengue vaccine and therapeutic trials and will help clarify behavioral responses to illness. To quantify heterogeneity in dengue illness, including milder cases, we developed the Dengue Illness Perceptions Response (IPR) survey, which captured detailed symptom data, including intensity, duration, and character, and change in routine activities caused by illness. During 2016–2019, we collected IPR data daily during the acute phase of illness for 79 persons with a positive reverse transcription PCR result for dengue virus RNA. Most participants had mild ambulatory disease. However, we measured substantial heterogeneity in illness experience, symptom duration, and maximum reported intensity of individual symptoms. Symptom intensity was a more valuable predicter of major activity change during dengue illness than symptom presence or absence alone. These data suggest that the IPR measures clinically useful heterogeneity in dengue illness experience and its relation to altered human behavior.

Dengue classically presents as an acute febrile illness lasting ≈5 days and accompanied by headache, musculoskeletal pain, and rash (*1*). A minority of infected persons show development of plasma leakage syndrome, intravascular volume loss, or major bleeding, which can lead to shock and death (*2*). There are 4 serotypes of dengue (DENV), and persons show development of long-lasting immunity to the specific serotype after infection. Cross-reactive immunity provides short-term protection against other serotypes. However, under some circumstances, previous infection with a different serotype increases the risk for severe disease (*3*). The World Health Organization (WHO) classification of dengue focuses on distinguishing between mild cases (classic dengue) and persons with or at risk for major adverse outcomes or death (severe disease) (*4*–*6*). Moreover, most literature describing the clinical manifestations of dengue evaluates the healthcare-seeking population whose symptoms are likely to be more severe. DENV infections associated with milder illness have not been subjected to similar systematic analysis or characterization.

Although the focus on severe disease is an obvious priority, there is value to characterizing the subjective illness experience in persons who have milder disease. As dengue vaccine development evolves, one of the challenges will be to accurately measure the effect of vaccination on the severity of illness. Measuring the rates of severe disease in vaccine trials is an insensitive approach and addresses only the small fraction of cases meeting these criteria, leaving open the possibility that vaccinated persons not meeting the criteria for severe disease had a meaningfully different disease experience than unvaccinated persons. Behavioral responses and reactions to illness depend on the illness experience of a person and will determine whether they attend work or school, self-medicate, seek medical attention, and move around their neighborhood, potentially infecting mosquitoes at other sites (*7*). Quantifying these relationships will help identify the human factors essential for virus transmission, guide the design of improved control strategies, assist policy makers in assessing the burden of dengue illness and healthcare needs, and guide allocation of resources.

As part of a larger epidemiologic study we developed the dengue Illness Perceptions Response (IPR) survey to gather data to characterize the dengue illness experience of a person, including the range and intensity of symptoms, and to measure the response of a person to their illness. We outline the development and application of the IPR, and to illustrate its potential value, we describe and quantify the heterogeneity of dengue illness and its associations with behavior changes.

## Methods

### Ethics

The study protocol was approved by the Naval Medical Research Unit No. 6 (NAMRU-6) Institutional Review Board (IRB) (protocol #NAMRU6.2014.0028) in compliance with all applicable federal regulations governing the protection of human subjects. IRB relying agreements were established between NAMRU-6, the University of California Davis, Tulane University, Emory University, and the University of California, San Diego. The protocol was reviewed and approved by the Loreto Regional Health Department, which oversees health research in Iquitos.

### Field Site

We conducted the study in an established research unit in the Amazonian city of Iquitos, Peru (*7*–*9*). Based in the department of Loreto, Iquitos has a population of ≈400,000 and mostly relies on tourism and extractive industries (*8*,*10*). More than half of the population of Loreto depend on government health insurance, which is available for persons living in poverty (*11*). Dengue is endemic to Iquitos, and 1 serotype typically dominates at any one time; all 4 DENV serotypes have circulated in Iquitos over the past 3 decades (*8*). The force of infection for DENV in Iquitos was calculated to vary from 0 to 0.33 infections/susceptible person/year during 1999–2010 (*12*). In March 2016, Zika virus was detected in Iquitos, and its transmission dominated for ≈1.5 years (*13*). Since 2017, the Asian-American strain of DENV-2 has been the dominant circulating serotype (A.C. Morrison, unpub. data).

### Development of IPR Survey

On the basis of the experience of our team in collecting dengue symptom data and the available literature, we developed a focus group guide used to facilitate 6 mixed-sex focus groups to assess how persons who had recently had dengue illness (or an adult family member of a child who was infected) described the experience, including the range, duration, and precise location of symptoms; ways to describe the severity of the symptoms; and word choices related to the symptoms. Focus group participants (n = 52) were persons who had laboratory-confirmed dengue (positive result on DENV reverse transcription PCR [RT-PCR]) during the previous 3 months and who were recruited by ongoing community or clinic-based febrile illness surveillance. Using the range of symptoms and descriptions elicited through the focus groups, the research team developed a first version of the IPR, which was reviewed by collaborating experts and 3 local clinicians experienced in managing dengue to ensure its medical relevance. These data informed the IPR development, helping to define the symptoms to be included and descriptive terms used for symptoms and determine how to measure symptom intensity (there was almost unanimous support for scales using facial expressions to grade intensity).

The IPR survey that was implemented collected data on 36 symptoms; depending on the specific symptom, these data included presence, duration, intensity, character, frequency and location of symptoms (Appendix Table 1). We learned about descriptive terms for various symptoms: musculoskeletal pain was most commonly described as “beaten up,” affecting the whole body. Headaches were most commonly described as a “generalized pressure.” Abdominal pain was most commonly described as “cramping” and most frequently located in the epigastrium. The survey also asked to what extent symptoms had affected daily activities: no change, minor change, or major change. The IPR was then piloted on 54 persons: 7 children <10 years of age, 10 persons 10–20 years of age, and 37 persons >20 years of age. Feedback from this pilot testing was used to guide final modifications of the survey; data from these persons are not included in the main analyses.

### Study Design

The study followed a contact-cluster design. Persons positive for serum DENV RNA by RT-PCR (index case-participants) were identified through community- or clinic-based febrile illness surveillance (*7*,*14*). At the time of blood collection, we administered a retrospective movement survey to the index case-participants to identify locations visited in the previous 15 days. As soon as the initial PCR result was available, usually the next weekday, persons (contacts) from the home of the index case-participants and any residential locations visited by the index case-participants were then invited to provide a blood sample, regardless of the presence of symptoms; we tested consenting persons for serum DENV RNA by using RT-PCR. The protocol enabled requesting follow-up samples from PCR-negative contacts at intervals of no less than 2 days; we tested a median of 2 (interquartile range 2–3) blood samples from contacts by using PCR.

Index case-participants and any contacts with positive RT-PCR results (*15*) for DENV RNA were invited to respond to a series of surveys relating to symptoms (IPR), movements throughout the city, health related qualify of life, and illness-related expenditures. The IPR survey was applied daily (where possible), starting from the day of the positive RT-PCR result until there were no reported symptoms for 2 days, and then again 30 days later. Inclusion criteria for the study were an age >5 years, DENV viremia documented by RT-PCR, and willingness to provide informed consent or assent for persons 5–17 years of age.

### Data Analysis and Statement

We used CommCare (https://www.dimagi.com), an open-source software platform, to develop a digital version of the IPR, which we administered by using handheld tablets (*16*). Survey data were uploaded to CommCare secure server where it could be reviewed by senior project members for discrepancies and corrected if necessary. We forwarded data to a PostgreSQL database (https://www.postgresql.org) and directly accessed this database and analyzed the data by using R version 3.5.1 (*17*). We categorized symptoms into the following clinically defined groups: constitutional, fever, headache, musculoskeletal, abdominal, cutaneous, respiratory, bleeding, and other (Appendix Table 2). The final dataset included the first 14 days of illness for each participant, indicating for these days symptom intensities from 0 (absence) to 10 (most intense). The survey solicited the maximum symptom intensity experienced between the day of collection and either the day the symptom started (in the first IPR) or the previous survey (in subsequent IPRs). Intensities recorded in the first IPR were assigned to the first date that the specific symptom was reported and any gaps in intensity data were imputed with linear interpolation. For days after the final survey, intensities and frequencies of symptoms were assumed to be 0. From this dataset we calculated the duration of illness and of specific symptoms, and the proportion of symptoms that were reported on each day of illness. Suspected dengue was defined following the 2009 WHO guidelines as fever and >2 of the following symptoms: headache, retroorbital pain, nausea/vomiting, muscle/joint pains, and rash (*4*).

We performed a correlation analysis of symptom intensities, excluding imputed intensity values, by using the cor function in the stats package in R with use argument as pairwise.complete.observations and method argument as spearman to generate a correlation matrix and then plotted a heatmap and dendrogram derived from the correlation matrix by using the pheatmap function in the pheatmap package (*18*). To compare index and contact cases, we first compared the mean number of reported symptoms by using a 2-sided Student *t*-test, and then compared the proportions of persons reporting specific symptoms by using the Fisher exact test. Because there were 36 comparisons, we applied a Bonferroni correction to the α value.

To explore the relationship between major activity change and individual symptom intensity, we performed logistic regression by using the glm function in the stats package in R, designating major activity change (present or absent) as the dependent variable and symptom intensity, age, and sex as independent variables. To evaluate the benefit of collecting intensity data versus only symptom presence and absence, we performed 2 logistic regression models for each symptom by using major activity change as the response variable and either symptom intensity or binary symptom presence as the explanatory variable. We used the difference in the model Akaike Information Criteria (Δ-AIC) as a means to compare the 2 models, with a positive AIC favoring the use of intensity over presence or absence. All data and R code used for this analysis are available (https://github.com/hammoire/dengue_ipr).

## Results

We enrolled 79 persons who completed a total of 429 IPR surveys (median 5 surveys/person) (Table 1). A total of 55 persons were enrolled through febrile illness surveillance (index case-participants), 42 through community-based surveillance, and 13 through clinic-based surveillance. The remaining 24 persons were enrolled through cluster investigations (contact case-participants); these case-participants were identified from the total of 408 contacts tested (72% of the 567 eligible contacts). Index and contact case-participants were similar in age and sex. The first survey was completed a median of 3 days after the onset of symptoms (range −1 to 8 days). A total of 75% of participants completed the follow-up survey at a median of 36 days after the onset of symptoms (range 21–82 days). Seven (9%) participants were hospitalized during the course of their acute infection (Table 1).

**Table 1:** Baseline characteristics of participants tested for heterogeneity of dengue illness in community-based prospective study, Iquitos, Peru*

Characteristic	Total	Index	Contact
No. participants	79	55	24
No. surveys	429	309	120
Sex, no. (%)			
M	38 (48)	27 (49)	11 (46)
F	41 (52)	28 (51)	13 (54)
Median age, y (IQR)	17 (12–27.5)	17 (14–26)	14.5 (9.5–31)
Day at diagnosis (IQR)	3 (2–4)	4 (3–5)	2 (1–3)
Serotype, %			
DENV-2	76 (96)	53 (96)	23 (96)
DENV-3	3 (4)	2 (4)	1 (4)
WHO suspected dengue† (%)	67 (85)	51 (93)	16 (67)
Warning signs, no. (%)	20 (25)	18 (33)	2 (8)
Hospitalized, no. (%)	7 (9)	6 (11)	1 (4)

*DENV, dengue virus; IQR, interquartile range; WHO, World Health Organization. †Persons who met the 2009 WHO criteria for suspected dengue (see Methods).

### Frequency and Duration of Symptoms

We summarized the overall frequency (Appendix Figure 1) and duration (Appendix Figure 2) of symptoms. The frequency of individual symptoms did not differ by sex or age group (younger vs. older than 18 years of age). All symptoms occurred more frequently in index case-participants than in contact case-participants, with the exception of vaginal bleeding. These differences were only significant for bad taste and chills (p<0.01 with Bonferroni correction) (Appendix Table 3).

Participants reported a mean symptom duration of 7.37 days. One person (a contact case-participant) experienced no symptoms. A total of 5 persons experienced >1 symptoms between the follow-up visit and the last form in the acute phase of illness, including nausea (2), malaise (2), headache (1), congestion (1), itching (1), and fainting (1).

### Timing and Characterization of Symptoms

**Figure 1:**
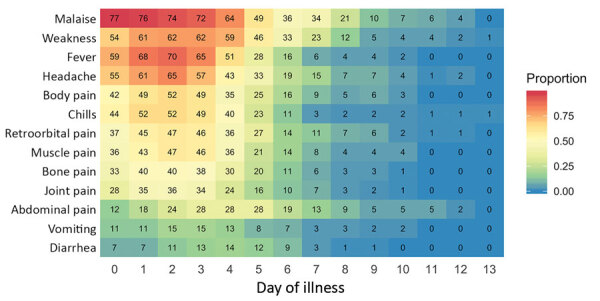
Timing of 13 key dengue symptoms for participants tested for heterogeneity of dengue illness in community-based prospective study, Iquitos, Peru. The x-axis represents day of illness and y-axis individual symptoms. Numbers in tiles indicate total number of persons with a symptom on that day. A total of 79 persons infected with dengue virus participated in surveys.

We report the timing of each of 13 symptoms for which duration data were collected (Figure 1). Malaise preceded other symptoms by 1 day in a substantial fraction of cases and was still reported by >30% of persons at day 7. Fever, headache, and pain (body/muscle/bone/joint) were most frequently reported on days 1–3, whereas abdominal pain was most frequently reported on days 3–5 (Figure 1).

**Figure 2:**
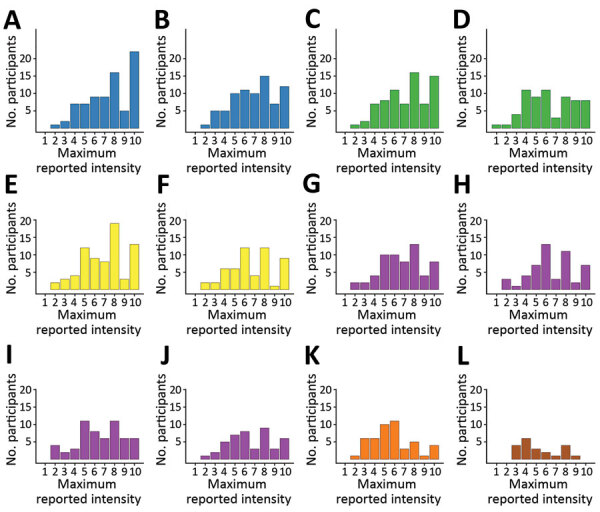
Histograms of maximum reported symptom intensities for participants tested for heterogeneity of dengue illness in community-based prospective study, Iquitos, Peru. Persons who did not report symptoms were excluded. Colors in histograms correspond to symptom groups defined in Appendix Figure 1. Values for each panel are no. (%) of participants who reported the specific symptom at any time during their illness. A) malaise, 78 (98.7); B) weakness, 76 (96.2); C) fever, 74 (93.7); D) chills, 65 (82.3); E) headache, 72 (91.1); F) retroorbital pain, 54 (68.4); G) body pain, 61 (77.2); H) bone pain, 51 (64.6); I) muscle pain, 57 (72.2); J) joint pain, 45 (57.0); K) abdominal pain, 47 (59.5); L) sore throat, 21 (26.6).

We found substantial heterogeneity in reported maximum intensity per symptom by participants. We compiled the distribution of the maximum intensity reported by each person during the illness period for 12 key symptoms on a 10-point scale (Figure 2). Symptoms with the highest median values for maximum intensity (excluding those that did not report the symptom at all) were malaise and fever (*8*), body pain, headache, muscle pain, and weakness (*7*) (Figure 2).

**Figure 3:**
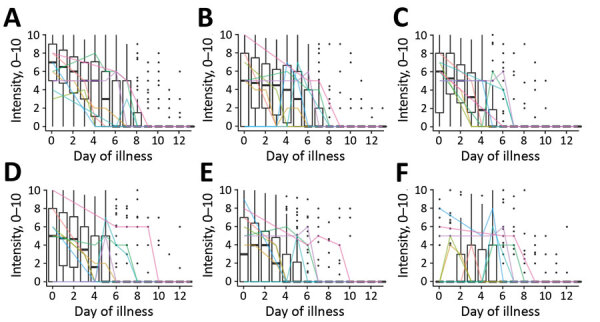
Symptom intensities (scale 1–12) for 6 symptoms over the first 14 days of illness (0–13) for participants tested for heterogeneity of dengue illness in community-based prospective study, Iquitos, Peru. A) Malaise; B) weakness; C) fever; D) headache; E) body pain; F) abdominal pain. Box plots indicate trends for the study population as a whole. Dark horizontal lines indicate median, upper limit of box indicates 75th percentile, lower limit of box indicates 25th percentile, upper whisker extends to the largest value <1.5 times the interquartile range; and lower whisker extends to the smallest value >1.5 times the interquartile range. Black dots indicate individual scores. Colored lines indicate trajectories for a random sample of 10 individual participants.

We report the trajectories of symptom intensity over the course of the illness for 6 symptoms (Figure 3); if the symptom was absent, an intensity of 0 was assigned. For the study population as a whole, the intensity of individual symptoms followed a similar timing as the presence or absence of each symptom. However, there was substantial variation in the trajectories of symptom intensity by participant (Figure 3).

**Figure 4:**
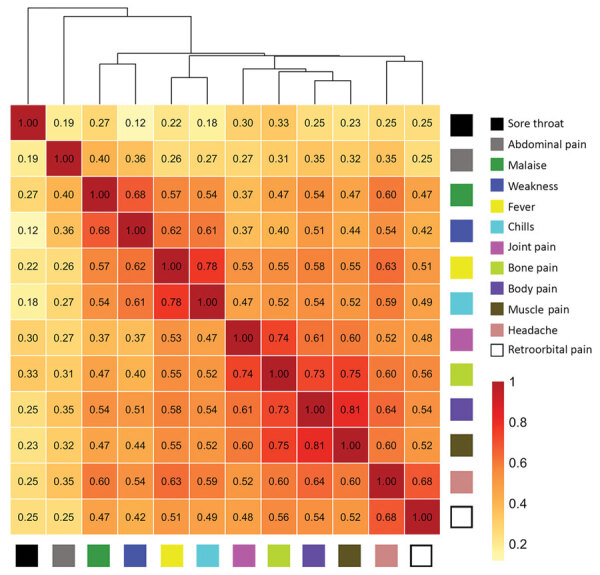
Correlations of intensities of individual symptoms (379 surveys, 79 participants) and hierarchical clustering for participants tested for heterogeneity of dengue illness in community-based prospective study, Iquitos, Peru. Tile colors indicate strength of correlations. The height at which symptoms are linked in the dendrogram indicates how strongly they are related (lower height indicates a closer link).

We report correlations between the intensities of individual symptoms and the hierarchical clustering dendrogram of symptom intensities (Figure 4). Pairwise correlations ranged from 0.12 (sore throat vs. weakness) to 0.81 (body pain vs. muscle pain). Symptom intensity scores clustered into distinct groups (i.e., constitutional [malaise and weakness], fever/chills, headache/retroorbital pain, and musculoskeletal [body, muscle, bone, and joint pains]). Abdominal pain and sore throat did not cluster with other symptoms in this analysis.

### Symptom Intensity and Activity Change

A total of 48 (61%) participants reported a major change in their daily activities on >1 days, 25 participants (32%) reported a minor change in daily activities, and only 6 participants (8%) reported no change in daily activities during their illness. On the basis of logistic regression models analyzing major activity change as a function of individual symptom intensities, corrected for age and sex, weakness had the strongest association with major activity change (odds ratio 1.48, 95% CI 1.36–1.63), followed by malaise (odds ratio 1.36, 95% CI 1.25–1.48) (Table 2).

**Table 2:** Effect of symptom intensity on reporting of major activity change for participants tested for heterogeneity of dengue illness in community-based prospective study, Iquitos, Peru

Symptom	Odds ratio (95% CI)*	Δ-AIC†
Malaise	1.36 (1.25–1.48)	42.1
Weakness	1.48 (1.36–1.63)	35.8
Fever	1.28 (1.19–1.38)	20.8
Abdominal	1.34 (1.22–1.48)	9.9
Body pain	1.32 (1.22–1.43)	9.1
Headache	1.27 (1.17–1.37)	9.0
Chills	1.31 (1.21–1.43)	3.3
Muscle pain	1.25 (1.15–1.35)	2.7
Joint pain	1.23 (1.13–1.34)	−0.0
Retroorbital pain	1.11 (1.03–1.2)	−0.1
Sore throat	1.02 (0.87–1.18)	−0.5
Bone pain	1.24 (1.15–1.36)	−1.4

*Shown is the increase in odds of reporting a major activity change when symptom intensity is increased by 1 point for each of the 12 symptoms.  †Δ-AIC, difference in Akaike Information Criteria between models by using binary symptom presence versus symptom intensity (0–10) as a predictor of major activity change. A positive Δ-AIC favors the use of intensity over presence of symptom alone.

To assess the added value of measuring symptom intensity versus only symptom absence or presence, we compared logistic regression models that used major activity change as the dependent variable (compared with minor or no activity change as reference) and either the presence of a symptom or the symptom intensity as the independent variable. The difference in the model (Δ-AIC) was used to compare the 2 models, in which a positive Δ-AIC would favor the use of symptom intensity over presence/absence alone. Symptoms with the greatest positive Δ-AIC were malaise (Δ-AIC 42.1), weakness (Δ-AIC 35.8), and fever (Δ-AIC 20.8) (Table 2). These data indicate that symptom intensity is more valuable than symptom presence or absence alone as a predictor of major activity change during DENV infection.

## Discussion

Symptoms reported most frequently by study participants were consistent with classical descriptions of dengue illness, other cohort studies, and WHO guidelines, as well as the key symptoms reported by participants in our focus groups (*19*–*22*), although a large fraction of participants reported less typical gastrointestinal or respiratory symptoms. Participants enrolled as contact case-participants reported fewer symptoms, similar to the findings in cluster investigations performed in Thailand (*23*). Some of these persons might otherwise not have sought medical attention. Only 1 (contact case-participant) person reported no symptoms. This finding conflicts with evidence suggesting that only 12%–25% of DENV infections are apparent (*24*,*25*). However, it is likely that administering the IPR survey encouraged reporting of symptoms that would not have been recalled in the context of a retrospective questionnaire, possibly explaining the relatively high symptomatic to asymptomatic ratio in our sample. We only collected data for persons who had a positive RT-PCR result (persons with only immunologic evidence of seroconversion to DENV were not included). This limitation has been associated with a higher frequency of symptoms (*23*).

Although most persons had mild dengue illness, our data demonstrate a range of intensity levels for individual symptoms. The IPR survey also enabled us to identify symptom groups within which daily intensities were highly correlated (r >0.70): constitutional (malaise and weakness), fever (fever and chills), headache (headache and retroorbital pain), and musculoskeletal pain (body, muscle, bone, and joint pain).

Abdominal pain was reported by 59% of participants, but only 13% reported severe abdominal pain (intensity >6). Abdominal pain followed a somewhat different time course from and showed substantially lower correlation with the symptom groups listed above. This finding is consistent with a distinct physiologic mechanism for abdominal pain. It also supports guidelines classifying severe abdominal pain as a warning sign, although few of our participants had evidence of plasma leakage or severe bleeding.

Although our study population consisted primarily of persons with mild dengue illness, participants still reported a substantial impact of illness on daily activities. Use of intensity scores for the major symptoms substantially improved the assessment of the effects of illness on daily activities when compared with use of symptom presence/absence alone. Therefore, the ability of the IPR survey to capture this aspect of heterogeneity in nonhospitalized persons with dengue could help improve assessment of either beneficial or detrimental effects of interventions, as proposed by Thomas et al. (*26*). Moreover, the causes of specific symptoms in dengue remain poorly defined (*19*); instruments such as the IPR survey could potentially be used to explore these underlying mechanisms.

Recently, a group of experts proposed a data collection tool to capture the overall experience of a person with dengue based on how their symptoms affect general wellness and functionality (*26*). The Dengue Illness Index (DII) records the presence or absence of symptoms daily. Our IPR survey has similarities to the DII, but a major difference is that the IPR solicited the assessment of the intensity of key symptoms of a participant. Our data suggest that persons are able to provide such an assessment and that intensity data add information relevant to the overall assessment of illness impact.

Thomas et al. (*26*) proposed a strategy for tabulating the DII to yield a single illness score. We did not assign weights a priori for the different symptom intensities. Our data showing high correlations within symptom groups suggests that each symptom should not be given equal weight. We are exploring approaches to express the symptom severity data to a single or small number of the most informative parameters (e.g., principal component analysis). Regardless of the specific approach used to score dengue symptom severity, it will be essential to define the relationships of severity score to other external measures of illness impact. In addition to data on change in daily activities, described here, persons also provided data on movement (*14*) and on a health-related quality of life survey, which we are incorporating into future analyses.

Our findings should be interpreted in light of several additional limitations. The IPR survey was administered to participants by research staff using a tablet-based application. Some choices in the design of the tablet-based survey addressed operational needs or preferences of the research team. These considerations created some unanticipated challenges and required minor modifications to the tool during the course of our study. For example, as a result of delays in receiving RT-PCR results or missed follow-up assessments, it was difficult to accurately assign a start and end date of some symptoms for some persons. Imputation of missing data introduces error into our dataset that is difficult to quantify. Our sample size is relatively small and homogenous in host and viral populations. Our study was focused on evaluation of persons with acute DENV infection and did not include participants with nondengue febrile illnesses for comparison. Persons who participated in the focus groups or the main study are not representative of the overall population of Iquitos (e.g., greater time availability or willingness to engage with medical personnel). That said, the instrument development process, which engaged participants recently given a diagnosis of DENV infection and clinical experts who reviewed the literature, resulted in a tool that assessed a wide range of symptoms and potential behavioral responses, which we believe could be applied in other settings, although piloting the tool before use elsewhere is advisable. Febrile illness surveillance was limited to selected neighborhoods, and contacts were identified on the basis of social proximity. Given the long-standing interactions of the research team with the local population and the demographics of study participants, we do not expect these considerations to have introduced major bias in our results.

Our data support the feasibility and rationale of efforts to quantify dengue illness in future natural history and intervention studies. Our experience should be useful to guide development of reliable and validated tools for this purpose. We anticipate that the IPR survey could be adapted to other formats, including self-administration by research subjects, and to other languages, but these efforts would require modifications and further validation. Further studies are needed to test our results across other populations, to assign appropriate weights to individual symptom scores, and to correlate with other biologic and epidemiologic measures of disease impact.

Appendix Additional information on heterogeneity of dengue illness in community-based prospective study, Iquitos, Peru.
