# Supplementary material for: Heterogeneity of Dengue Illness in Community-Based Prospective Study, Iquitos, Peru
Source: Emerg Infect Dis. 2020 Sep;26(9):2077–86. doi: 10.3201/eid2609.191472 (PMC7454099; doi:10.3201/eid2609.191472)
Supplement: Appendix — Additional information on heterogeneity of dengue illness in community-based prospective study, Iquitos, Peru. [file 19-1472-Techapp-s1.pdf]

Article DOI: https://doi.org/10.3201/eid2609.191472

Heterogeneity of Dengue Illness in Community-Based Prospective Study, Iquitos, Peru

Appendix

Appendix Table 1. Description of data collected for symptoms of dengue illness on the Illness Perceptions Response form for participants tested for heterogeneity of dengue illness in community-based prospective study, Iquitos, Peru*

Symptom	Presence	Duration	Intensity	Frequency	Time of day	Character	Pattern	Alleviating factor	Location
Malaise	✓	✓	✓	–	–	–	✓	✓	–
Fever	✓	✓	✓	✓	–	–	✓	✓	–
Chills	✓	✓	✓	✓	–	–	✓	✓	–
Weakness	✓	✓	✓	–	–	–	–	–	–
Anorexia	✓	–	–	–	–	–	–	–	–
Headache	✓	✓	✓	–	✓	✓	✓	✓	✓
Retroorbital pain	✓	✓	✓	–	–	–	–	–	–
Body pain	✓	✓	✓	–	✓†	✓†	✓†	✓†	✓†
Bone pain	✓	✓	✓	–	✓†	✓†	✓†	✓†	✓†
Joint pain	✓	✓	✓	–	✓†	✓†	✓†	✓†	✓†
Muscle pain	✓	✓	✓	–	✓†	✓†	✓†	✓†	✓†
Abdominal pain	✓	✓	✓	–	–	✓	✓	–	✓
Nausea	✓	–	–	–	–	–	–	–	–
Vomiting	✓	✓	–	✓	–	–	–	–	–
Diarrhea	✓	✓	–	✓	–	✓	–	–	–
Petechiae	✓	–	–	–	–	–	–	–	–
Purpura	✓	–	–	–	–	–	–	–	–
Hematemesis	✓	–	–	–	–	–	–	–	–
Melena	✓	–	–	–	–	–	–	–	–
Gun bleeding	✓	–	–	–	–	–	–	–	–
Nasal bleeding	✓	–	–	–	–	–	–	–	–
Blood in urine	✓	–	–	–	–	–	–	–	–
Vaginal bleeding	✓	–	–	–	–	–	–	–	–
Congestion	✓	–	–	–	–	–	–	–	–
Sore throat	✓	–	✓	–	–	–	–	–	–
Cough	✓	–	–	–	–	–	–	–	–
Jaundice	✓	–	–	–	–	–	–	–	–
Maculopapular rash	✓	–	–	–	–	–	–	–	✓‡
Erysipelas	✓	–	–	–	–	–	–	–	✓‡
Bad taste	✓	–	–	–	–	–	–	–	–
Chest pain	✓	–	–	–	–	–	–	–	–
Sputum	✓	–	–	–	–	–	–	–	–
Photophobia	✓	–	–	–	–	–	–	–	–
Fainting	✓	–	–	–	–	–	–	–	–
Ear pain	✓	–	–	–	–	–	–	–	–
Itching	✓	–	–	–	–	–	–	–	–

*Alleviating factor, factor that helps manage symptoms; character, description of symptoms (includes option for other); duration, number of days ago that the symptom started (asked on the first survey); frequency, no. episodes in previous 24-hour period; intensity, symptom intensity on a scale of 10 based on the smiley face scale; location, location of pain/rash; pattern, comes and goes or constant; presence, presence or absence of these symptoms; time of day, time of day symptoms occurred: morning, afternoon, night (any combination). Checks indicate yes; –. Data not collected.
†Data was collected about the musculoskeletal group not the individual symptoms.
‡Location data was collected for both these rashes together not individually.

Appendix Table 2. Comparison of the total number of symptoms experienced by index and contact cases for participants tested for heterogeneity of dengue illness in community-based prospective study, Iquitos, Peru*

Symptom	Index, mean (SD)	Contact, mean (SD)	p value	Sig_95	Sig_99
All	16.1 (4.259)	11.1 (4.656)	5.81×10^{-5}	True	True

*Sig_95, significant at the 5% level; Sig_99, significant at the 1% level.

Appendix Table 3. Comparison of the proportion of participants experiencing each symptom for Index and Contact cases for participants tested for heterogeneity of dengue illness in community-based prospective study, Iquitos, Peru*

Symptom	Group	Index, no. (%)	Contact, no. (%)	p value	Sig_95	Sig_99
Bad taste	Other	41 (74.5)	6 (25)	5.43×10^{-5}	True	True
Chills	Fever	51 (92.7)	14 (58.3)	5.94×10^{-4}	True	False
Fever	Fever	55 (100)	19 (79.2)	0.00188592	False	False
Sore throat	Respiratory	21 (38.2)	2 (8.3)	0.00724521	False	False
Vomiting	Abdominal	29 (52.7)	5 (20.8)	0.01278489	False	False
Headache	Headache	53 (96.4)	19 (79.2)	0.02444751	False	False
Weakness	Constitutional	55 (100)	21 (87.5)	0.02559466	False	False
Photophobia	Headache	11 (20)	0 (0)	0.02866452	False	False
Itching	Cutaneous	31 (56.4)	7 (29.2)	0.03032907	False	False
Retroorbital pain	Headache	42 (76.4)	12 (50)	0.03405395	False	False
Fainting	Other	26 (47.3)	6 (25)	0.08283892	False	False
Gum bleeding	Bleeding	7 (12.7)	0 (0)	0.09445268	False	False
Cough	Respiratory	13 (23.6)	2 (8.3)	0.13202128	False	False
Erysipelas	Cutaneous	31 (56.4)	9 (37.5)	0.14711476	False	False
Chest pain	Other	10 (18.2)	1 (4.2)	0.15815388	False	False
Joint pain	Musculoskeletal	34 (61.8)	11 (45.8)	0.2218841	False	False
Anorexia	Constitutional	51 (92.7)	20 (83.3)	0.23674371	False	False
Malaise	Constitutional	55 (100)	23 (95.8)	0.30379747	False	False
Diarrhea	Abdominal	22 (40)	6 (25)	0.30633875	False	False
Hematemesis	Bleeding	5 (9.1)	0 (0)	0.31553323	False	False
Purpura	Bleeding	5 (9.1)	0 (0)	0.31553323	False	False
Abdominal pain	Abdominal	35 (63.6)	12 (50)	0.32124216	False	False
Maculopapular rash	Cutaneous	10 (18.2)	2 (8.3)	0.32761681	False	False
Petechiae	Bleeding	7 (12.7)	1 (4.2)	0.42342415	False	False
Nausea	Abdominal	38 (69.1)	14 (58.3)	0.44086771	False	False
Bone pain	Musculoskeletal	37 (67.3)	14 (58.3)	0.45571627	False	False
Vaginal bleeding	Bleeding	1 (1.8)	1 (4.2)	0.51801363	False	False
Muscle pain	Musculoskeletal	41 (74.5)	16 (66.7)	0.58640912	False	False
Ear pain	Respiratory	5 (9.1)	1 (4.2)	0.66135318	False	False
Congestion	Respiratory	7 (12.7)	2 (8.3)	0.71490855	False	False
Body pain	Musculoskeletal	43 (78.2)	18 (75)	0.77594226	False	False
Blood in urine	Bleeding	3 (5.5)	1 (4.2)	1	False	False
Melena	Bleeding	2 (3.6)	0 (0)	1	False	False

*Sig_95, significant at the 5% level; Sig_99, significant at the 1% level.

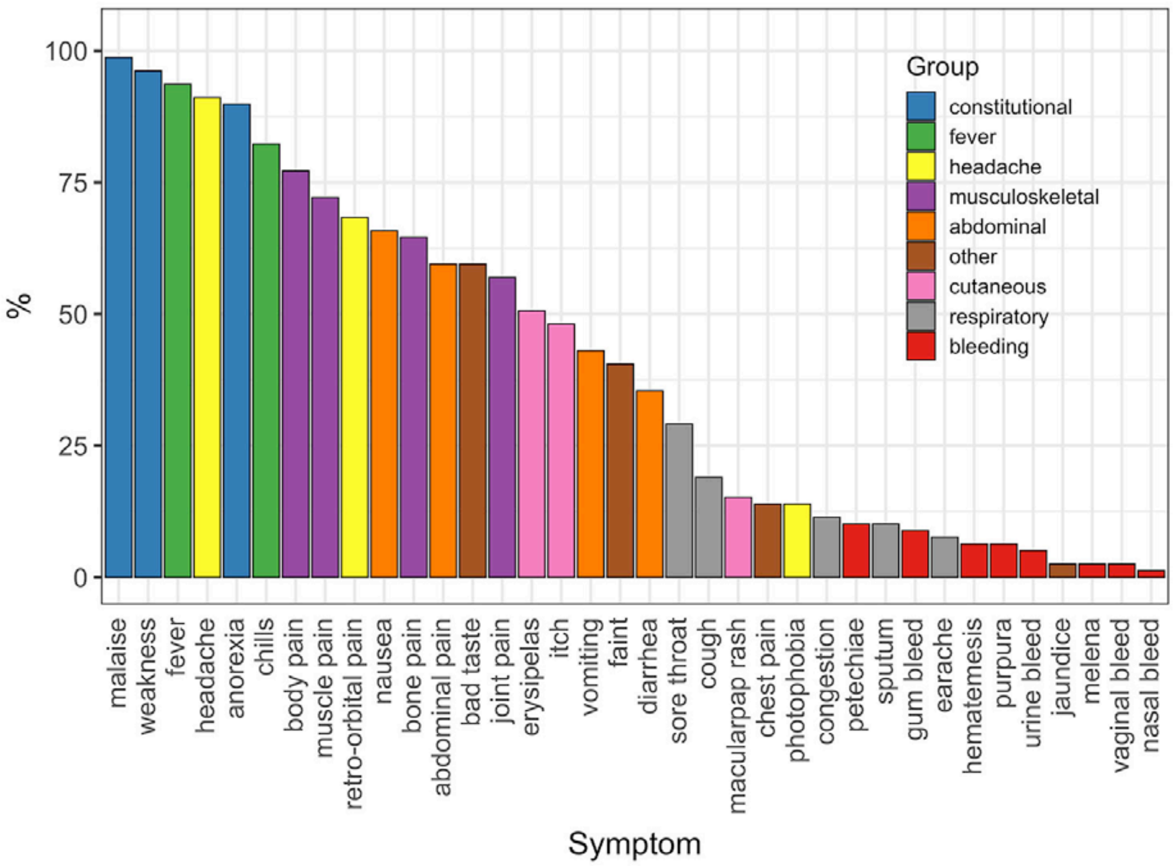

Appendix Figure 1. Percentages for 79 participants reporting specific symptoms during acute dengue illness during testing for heterogeneity of dengue illness in community-based prospective study, Iquitos, Peru Numbers above each bar indicate number of persons reporting the symptom. Colors represent clinically defined groups.

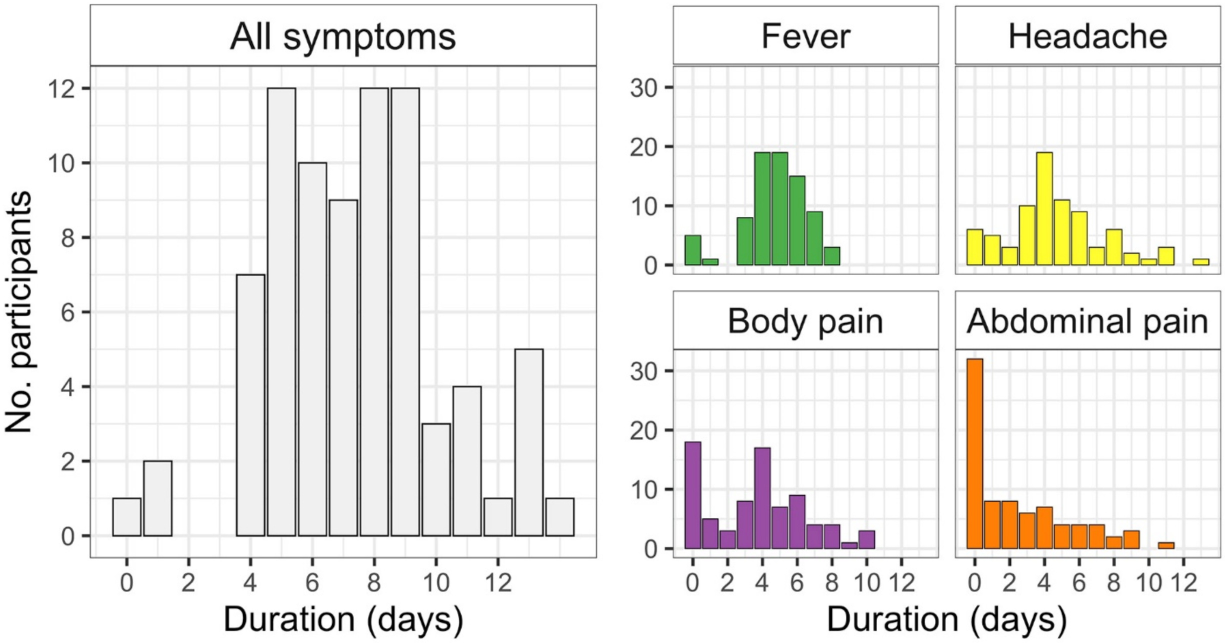

Appendix Figure 2. Histograms of duration (in days) of illness (any symptom) and specific symptoms experienced by participants who had dengue illness during testing for heterogeneity of dengue illness in community-based prospective study, Iquitos, Peru Colors in the right 4 panels indicate symptom categories defined in Figure 1.
